# Role of T Cells in Chikungunya Virus Infection and Utilizing Their Potential in Anti-Viral Immunity

**DOI:** 10.3389/fimmu.2020.00287

**Published:** 2020-02-21

**Authors:** Chek Meng Poh, Yi-Hao Chan, Lisa F. P. Ng

**Affiliations:** ^1^Singapore Immunology Network, Agency for Science, Technology and Research, Singapore, Singapore; ^2^National University of Singapore Graduate School for Integrative Sciences and Engineering, National University of Singapore, Singapore, Singapore; ^3^Department of Biochemistry, Yong Loo Lin School of Medicine, National University of Singapore, Singapore, Singapore; ^4^Institute of Infection and Global Health, University of Liverpool, Liverpool, United Kingdom

**Keywords:** chikungunya, T cells, immunopathology, vaccination, resident-memory T cells

## Abstract

Chikungunya virus (CHIKV) is an arthropod-borne alphavirus that causes hallmark debilitating polyarthralgia, fever, and rash in patients. T cell-mediated immunity, especially CD4^+^ T cells, are known to participate in the pathogenic role of CHIKV immunopathology. The other T cell subsets, notably CD8^+^, NKT, and gamma-delta (γδ) T cells, can also contribute to protective immunity, but their effect is not actuated during the natural course of infection. This review serves to consolidate and discuss the multifaceted roles of these T cell subsets during acute and chronic phases of CHIKV infection, and highlight gaps in the current literature. Importantly, the unique characteristics of skin-resident memory T cells are outlined to propose novel prophylactic strategies that utilize their properties to provide adequate, lasting protection.

## Introduction

Chikungunya virus (CHIKV) belongs to the *Togaviridae* family and is transmitted to humans by arthropod vectors, primarily the *Aedes aegypti* and *Ae. albopictus* mosquitoes within an urban cycle (1). This alphavirus was first isolated from a patient from Tanzania in 1952 (2), but it spread only to few regions before mellowing down to sporadic outbreaks in the next 30 years (3–11). It re-emerged in the last decade, starting from Kenya in 2004 (12, 13). Since then, it has broadened its geographical range to different regions of Africa, the Réunion island, Asia, Europe, and the Americas (4, 12, 14, 15).

CHIKV-infected patients develop chikungunya fever (CHIKF), a febrile illness characterized with acute hallmark polyarthralgia, along with other disease manifestations like fever and maculopapular rash (3, 16, 17). Symptoms usually manifest after 4–7 days of incubation period (3). CHIKV infection has been shown by multiple studies to induce robust immune responses. Specifically, the type-I interferon (IFN)-associated pathways (18–21), the recruitment of innate and adaptive immune cells to the site of infection (22), and the development of protective antibodies for virus resolution (23–29), has been shown to contribute significantly to the self-limiting nature of CHIKF. Although CHIKV-induced symptoms usually resolve in patients within 2 weeks (16), ~30–40% of these patients go on to develop chronic arthritis, which can be due to inefficient viral clearance, or persistent immune response in patients (3, 16, 18).

Host innate and adaptive immunity have multifaceted roles in CHIKV infection. While innate immunity in response to CHIKV infection has been well-studied (3, 17, 30), the functions of adaptive components, such as T cells and their myriad associated roles remain less defined. Recent studies have started to show that CHIKV-specific T cells and antibody response play significant roles in antiviral immunity, immunomodulation and pathology in CHIKV infection (24, 28, 31, 32). A better comprehension of the roles each T cell subset play during CHIKV infection may aid in understanding how to control disease progression and immune-mediated pathology. This review illustrates the significance of T cells in the protection and immunopathogenesis of acute and chronic arthritogenic disease. We also provide alternative perspectives on the prophylactic and therapeutic potential of T cells against CHIKV.

## CD8^+^ T Cells

**Figure 1:**
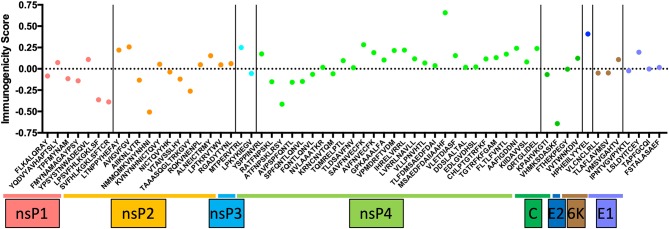
The list of conserved CD8 epitopes in CHIKV that was identified *in silico* by others and published in the literature were arranged according to the positions they occupy along the CHIKV proteome. The immunogenicity scores are determined through the Immune Epitope Database (IEDB) and plotted as shown. Of all the CD8 epitopes, only the three epitopes in the 6K region are experimentally validated.

CD8^+^ T cells have contrasting effects on alphavirus infection. In humans, CD8^+^ T cells express CD69, CD107a, granzyme B, and perforin during acute CHIKV infection (17, 33, 34), markers associated with T cell activation. Studies have identified putative CD8 epitopes within the CHIKV genome in mice and humans (Figure 1) (35, 36). Among these antigenic determinants, the non-structural proteins (nsP1-nsP4) contain a multitude of epitopes that can induce a robust immunological response. Only HLA-A24, B7, and B15 were predicted to express CD8 epitopes hidden within the capsid, E1 and E2 proteins (35). Despite the apparent abundance, only three HLA-A^*^0201 CD8 epitopes in CHIKV 6K protein were experimentally validated to trigger CD8 T cell response (37). The paucity of epitope validation highlights the inaccuracy of *in silico* modeling to predict epitope immunogenicity. Nevertheless, predicted epitopes require further testing to validate the sequences that are presented by different MHCs. Importantly, it remains unclear whether the recognition of CHIKV epitopes by CD8^+^ T cells has a role to play in eliminating virus-infected cells. This knowledge gap is worth investigating and will open up avenues to employ them as mediators in future CHIKV vaccines.

In mouse models, CD8^+^ T cell numbers in the joints increased during acute CHIKV infection. However, they do not protect against CHIKV-associated pathologies as mice deficient in CD8^+^ T cells still developed joint inflammation (31). This observation is in contrast to other findings whereby CD8 T cells play an active role in mediating viral clearance and disease resolution in mouse models of Ross River virus (RRV) (38) and Venezuela Equine Encephalomyelitis virus (VEEV) (39). CHIKV RNA could be detected in the footpad of mice at up to 16 weeks post-challenge, suggesting that chronic CHIKV infection could persist in the footpad (40). Persistent infection indicates that CHIKV epitopes could be continuously presented by antigen-presenting cells (APCs) to CD8^+^ T cells, leading to sustained activation of T cell receptor. This will eventually cause CD8^+^ T cell exhaustion and render them incapable of eliminating infected cells (41, 42), resulting in perpetual CHIKV infection. Indeed, the lower levels of CD8^+^ T cells and their decreased expression of activated marker CD69 suggest an exhausted phenotype in chronic CHIKV patients (33). In chronic viral (e.g., LCMV and HIV) and parasitic (e.g., malaria) diseases, persistent infection causes prolonged activation of CD8^+^ T cells, which eventually drives them to exhaustion and become unresponsive. This condition happens through, mostly but not limited to, the programmed death-1 signaling axis (43–46). The shutdown of exhausted CD8^+^ T cell function allows pathogens to remain established, cascading into a vicious cycle of a further immune shutdown and more persistent infection. Given that CHIKV infection is shown to persist in infected mice and individuals (34, 40), the CHIKV-specific CD8^+^ T cells may become exhausted and become incapable of controlling the infection. Targeting this pathway to reverse the exhaustion in CD8^+^ T cells has been demonstrated to eliminate chronic malaria infection in a mouse model (47), thus indicating that this is another potential avenue to treat chronic CHIKV infection.

## CD4^+^ T Cells

Activated CD4^+^ T cells have been well-documented to play an essential role in the pathogenesis of CHIKV-induced joint swelling (31, 34, 48). Our laboratory has comprehensively proven this through multiple methods. Mice depleted of CD4^+^ T cells do not develop joint pathology when challenged with CHIKV. Concordantly, the use of fingolimod, which blocks lymphocytes from exiting the lymphoid organs, also reduces joint pathology in CHIKV infected mice. In addition, TCR^−/−^ mice, which lack T cells, do not develop joint swelling when challenged (32). Independently, another group showed that the administration of antibodies against CTLA-4, which binds to CTLA-4 receptors found on the cell surface of activated CD4^+^ T cells, caused elimination of the latter and thereby reduced joint swelling in CHIKV-infected mice (49). Currently, there is no mouse model that develops the chronic arthralgia and arthritis as seen in chronic patients. Thus, it remains to be seen if the activated CD4^+^ T cells are also responsible for the chronic phase of CHIKV infection.

Pathogenic CHIKV-specific CD4^+^ T cells in CHIKV mouse models express T-bet and secrete IFN-γ, markers that are associated with Th1 phenotype (50). These findings concur with another study, which shows higher levels of Th1-associated cytokines in the sera of CHIKV-infected patients (34). Although this drives IFN-γ production during infection, the antiviral effects of IFN-γ are unable to block CHIKV replication in virus-infected cells due to CHIKV interference with downstream JAK-STAT signaling (51). These CD4^+^ T cells, together with CD8^+^ T cells, is detected in the joints of patients during the chronic phase of CHIKV infection (34). Two CHIKV CD4^+^ epitopes have been identified in mouse models thus far, with the cognate CD4^+^ T cells expressing IFN-γ during CHIKV infection (32). Despite CD4^+^ T cells being a cause of the pathogenic swelling accompanying CHIKV infection, they are also crucial in orchestrating the formation of CHIKV-specific antibody response. Dominant CHIKV-specific antibodies are of the isotype IgG2c (19, 28), and their production was severely impaired when CD4 T cells were absent (28).

## Regulatory T Cells

Among the heterogeneous CD4^+^ T cell population, regulatory T cells (Tregs) can also contribute to the protection against CHIKV. Tregs are able to interact with APCs in the peripheral lymph nodes and bring about downregulation of costimulatory molecules in the latter (52). Thus, when the APCs present CHIKV epitopes to CD4^+^ T cells, they become anergic instead and do not proliferate or migrate to the infected joints to cause joint swelling. However, the viral load in virus-infected mice was not affected by Tregs (52). Patients with acute and chronic CHIKV infections were shown to have lower levels of Treg, while recovered patients display Treg levels that are comparable to healthy controls (53). This finding concurs with the data from murine models, suggesting that Tregs prevent hyperactivation of pathogenic CD4^+^ T cells, thereby controlling joint pathology.

## Natural Killer T Cells

The impact of natural killer T (NKT) cells in CHIKV infection is less well-understood. In patients, acute CHIKV infection resulted in an increase of NKT cells in the blood (54). These NKT cells were found to upregulate the expression of IFN-γ, but not perforin. Although PBMCs from acute samples induced cytotoxicity in K562 cells during co-culture, it remains unclear if NKT cells account for the majority of the cytotoxicity effect (54). In chronic patients, NKT cell levels were significantly lower than healthy controls (55), although they still upregulated IFN-γ expression. In contrast, the expression levels of IFN-γ and TNF-α by NKT cells in convalescent patients are similar to that of healthy controls (54, 55).

In mice, levels of IFN-γ-expressing NKT cell remain unchanged in CHIKV-infected mice, as compared to healthy controls (48). However, these cells accumulate in the spleens of mice that are co-infected with CHIKV and malaria parasites (48). This event is due to the increased expression of chemokines CXCL9 and CXCL10 in the spleens, which attract and retain lymphocytes that express cognate chemokine receptors. Since mouse and human NKT cells express CXCR3 (which recognizes CXCL9 and CXCL10), these cells are likely to be trapped in the spleen, preventing them from migrating to the peripheral joints (56–58). Although this strategy of lymphocyte sequestration leads to decreased CHIKV immunopathology (32, 49), it is not clear how much does NKT cells contribute to this amelioration.

## Gamma-Delta (γδ) T Cells

Gamma-delta T cells are reported to play a protective role against CHIKV infection (59). These cells do not affect CHIKV virus replication in the joints. Instead, their absence is associated with increased joint swelling and tissue damage in the infected mice. The absence of γδ T cells were also linked to increased monocyte infiltration to the joints and increased levels of pro-inflammatory mediators CCL2, CXCL9, and IFN-γ (59). Hence, the resulting pro-inflammatory milleu leads to more inflammatory cell infiltration and result in tissue damage that is observed during CHIKV infection. However, there is no information on the role of γδ T cells in CHIKV patients. Thus, the extent of protection these cells offer in human settings remains unclear.

## Mobilizing T Cells Against CHIKV in Vaccine Design

**Figure 2:**
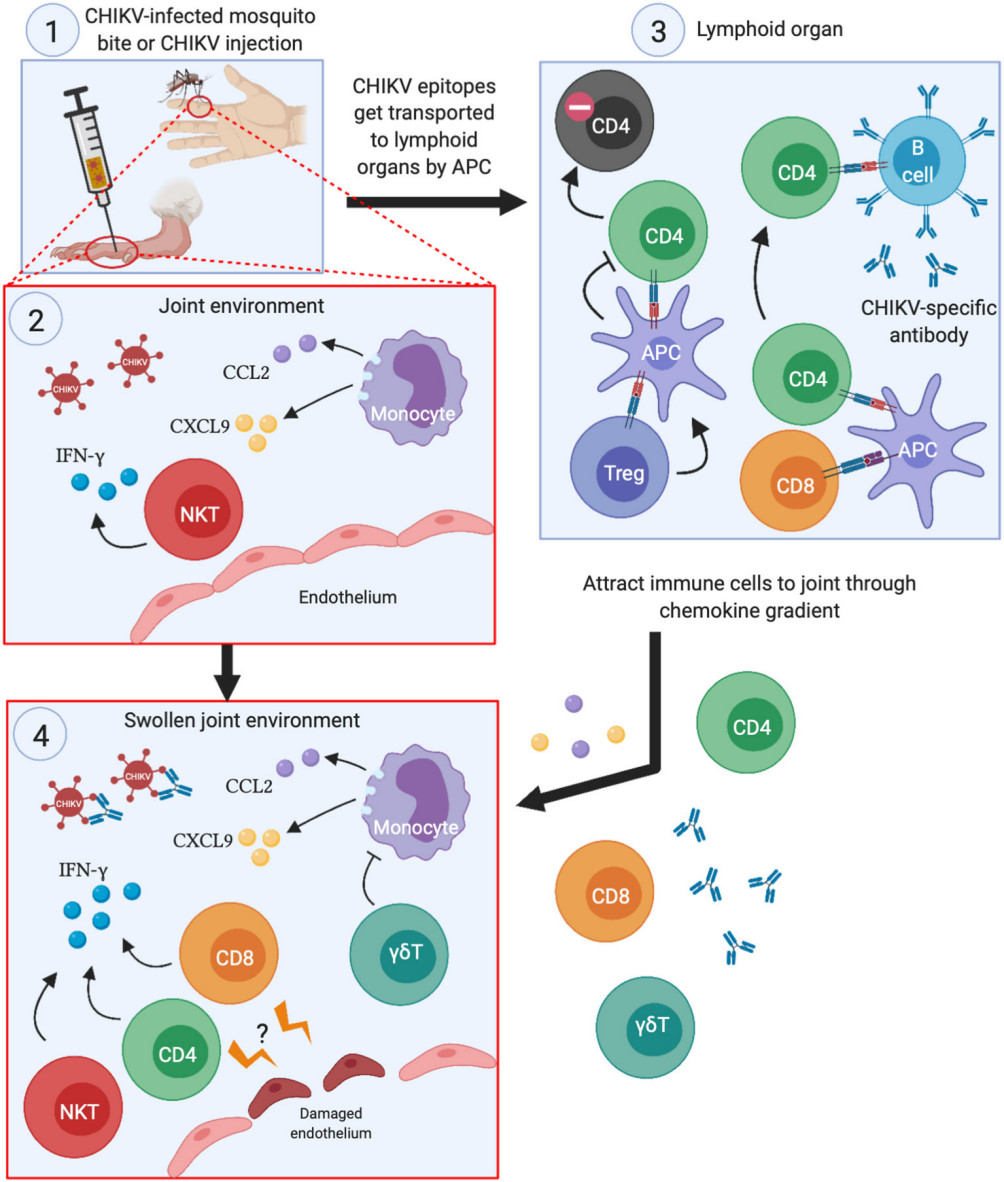
Summary of the roles each T cell subset play during acute CHIKV infection. (1) When CHIKV-infected mosquitoes transmit the virus to its human host (or experimental infection in mice), CHIKV infects the surrounding cells and creates pro-inflammatory milleu in the joints. (2) This serves to attract monocytes and NKT cells to the joints, which exacerbates the inflammation by secreting pro-inflammatory cytokines and chemokines, thereby potentiating the chemoattraction of other immune cells to the infection site. (3) The APCs that engulfs CHIKV antigens travel to lymphoid organs, present CHIKV epitopes to CD4 and CD8 T cells and activate them. Activated CD4 T cells then trigger CHIKV-specific B cells to develop antibodies that can neutralize the virus. Treg cells may also interact with APCs that ultimately lead to anergy in CHIKV-specific CD4 T cells. (4) Proliferating CD4 and CD8 T cells migrate to the swollen joints and contribute to the inflammation through IFN-γ secretion. Although not directly demonstrated, CHIKV-specific CD4 and CD8 T cells may recognize infected endothelial cells at the site of infection, leading to disruption of the barrier and worsens joint swelling.

The roles that each subset of T cells play in the joint swelling during CHIKV infection is illustrated in Figure 2. Despite their participation in CHIKV immunopathology, they have the potential to be included as mediators of effective anti-CHIKV vaccine strategies. Although researchers have been investigating experimental vaccines against CHIKV, most of these still rely on antibodies to neutralize the viruses (23, 28, 60–63). Furthermore, some of the CHIKV vaccines have been demonstrated to protect against other alphaviruses, such as RRV (19) and O'nyong'nyong virus (ONNV) (23). In the RRV challenge study, the lowest effective dose (0.1 μg virus) that conferred complete protection (denoted as the absence of viremia) did not increase the antibody response against CHIKV. Full protection only happens when the authors increased the dosage to 10 μg (19). This observation suggests that T cells may be instrumental in conferring protection at the lower doses. However, vaccination with recombinant baculovirus expressing CHIKV envelope proteins caused aged mice (>18 months old) to become more susceptible to CHIKV infection (62), which was partly explained by the lower antibody titers in the aged mice. Although antigenic variations among CHIKV isolates are not prevalent currently, subtle changes in the CHIKV proteome was demonstrated to affect the neutralizing effect of pre-existing anti-CHIKV antibodies (26, 64, 65). Thus, any mutation that results in reproductive-competent virus and yet abolishes antibody binding to CHIKV epitopes may result in future pandemic outbreaks.

## Utilizing Skin Resident Memory T Cells to Confer Pan-Alphavirus Immunity

The notion of inducing CHIKV-specific memory T cells as mediators of protection is attractive as T cell epitopes are generally better conserved than B cell antigens, mitigating the risk of immune escape through antigenic variations (66–69). Of the memory T cells, tissue-resident memory T cells (TRMs) are a unique subset of cells that is distinct from conventional memory T cells, in that the former dwell at the peripheral organs (70–73). The latter either reside in the lymphoid organs (central memory T cells) or recirculate in the body (effector memory T cells) (74, 75). Thus, TRMs could function as adaptive immune cells that act as first-line responders at the site of infection (71, 73, 76). Putative CHIKV-specific skin TRMs can be well-positioned to react to CHIKV-infected cells at the feeding site by virus-infected mosquitos during a blood meal.

Establishment of TRMs in the skin begins when clonally expanded T cells migrate from lymphoid organs to the skin. Some of these skin-homing T cells remain in place and develop into skin TRMs (77, 78). Vaccination leads to the widespread establishment of TRMs throughout the body, not only at the vaccination site (78). Thus, it is highly plausible that one can generate CHIKV-specific skin TRMs through vaccination at the skin. Interestingly, effector T cells were reported to migrate to all parts of the skin instead of accumulating only at the site of the infection or vaccination (79, 80). This phenomenon occurs due to the presence of E-selectin, ICAM-1 and other chemokines that are expressed by post-capillary venules at low levels throughout the skin (81). Hence, the resulting skin TRMs generated can protect against CHIKV infection from anywhere, regardless of where the virus-infected mosquitoes choose to probe and release the virus into the host.

In the event where CHIKV penetrates the skin barrier and infect the surrounding cells during blood feeding by mosquitoes, CHIKV-specific skin resident memory T cells can recognize virus-infected cells quickly. This identification leads to rapid coordination of the anti-CHIKV immune responses and leads to the elimination of virus-infected cells before the disease is established (82, 83). Moreover, CHIKV exposure should further boost pre-existing anti-CHIKV immune response and lead to the accumulation of more CHIKV-specific skin resident memory T cells (79, 80). Over time, repeated exposure of CHIKV should lead to further CHIKV-specific skin TRM accumulation all over the skin, with no establishment of CHIKV pathology.

The geographical range of mosquitoes that transmit the alphaviruses is projected to increase in the future due to climate change (84). Moreover, CHIKV shares some homology with other alphaviruses with the potential to cause unprecedented outbreaks (85–89). This shift may ultimately lead to the spread of CHIKV and other alphaviruses and cause future epidemics. However, as depicted by Roundy et al. (90) and Powers et al. (91), the close homology between CHIKV and other viruses within the family of alphaviruses suggest that they may share conserved epitopes that can induce T cell responses. Hence, some of the CHIKV-specific memory T cells can have the potential to target other alphaviruses and eliminate them. This knowledge may lead to the development of pan-alphavirus vaccines that are effective in preventing sudden alphavirus outbreaks, such as the spike in deaths from Eastern Equine Encephalomyelitis Virus (EEEV) in the United States in 2019, which is becoming endemic in the United States (92).

## Concluding Remarks

The re-emergence of CHIKV underscores the need for effective vaccine strategies to prevent further global CHIKV dissemination. In immunological naïve individuals, CHIKV infection puts them at risk of having their immune system manipulated toward CHIKV pathology and virus persistence. An optimal vaccine against CHIKV invokes not only humoral response, but more importantly, localized CHIKV-specific, cell-mediated immunity at the skin, and yet avoids the induction of CHIKV-mediated immunopathology that is associated with pathogenic CD4 T cells. By inducing anti-CHIKV TRMs at the skin before infection strikes, they can rapidly clear the virus before it disseminates and triggers the associated immunopathology. Besides, anti-CHIKV TRMs have the potential to react to more conserved CHIKV epitopes (which may also be conserved in closely related alphaviruses) and the immunity buildup increases with each natural infection, providing a robust, potent immunity against CHIKV, and possibly other alphaviruses.

## Author Contributions

All authors listed have made a substantial, direct and intellectual contribution to the work, and approved it for publication.

### Conflict of Interest

The authors declare that the research was conducted in the absence of any commercial or financial relationships that could be construed as a potential conflict of interest.
